# S-phase fraction and survival benefit from adjuvant chemotherapy or radiotherapy of breast cancer.

**DOI:** 10.1038/bjc.1994.483

**Published:** 1994-12

**Authors:** O. Stål, L. Skoog, L. E. Rutqvist, J. M. Carstensen, S. Wingren, S. Sullivan, A. C. Andersson, M. Dufmats, B. Nordenskjöld

**Affiliations:** Department of Oncology, Faculty of Health Sciences, Linköping University, Sweden.

## Abstract

Cancer chemotherapy interacts with cell proliferation, but data on the relationship between cancer cell replication and the effect of adjuvant chemotherapy are scarce. We have investigated the S-phase fractions of the primary tumour from premenopausal breast cancer patients who participated in a randomised trial comparing 12 cycles of polychemotherapy (CMF) with post-operative radiotherapy. DNA flow cytometry was performed on frozen tissues from 208 primary breast carcinomas, of which the S-phase fraction was estimated in 176 cases. There was a significantly higher benefit from CMF among patients with a high S-phase fraction (P = 0.0033). The relative risk of distant recurrence or death in the chemotherapy group as compared with the radiotherapy group was 0.19 for patients whose tumours had an S-phase fraction of 10% or over (95% CI 0.07-0.51) and 1.55 (0.88-2.73) for patients whose tumours showed lower S-phase levels. The interaction was still significant in multivariate analysis (P = 0.0057), including lymph node metastases, tumour size and oestrogen receptor content. We conclude that the benefit from adjuvant chemotherapy compared with radiotherapy is largely confined to patients with highly proliferative tumours.


Br. J. Cancer (1994), 70, 1258–1262 © Macmillan Press Ltd., 1994

### S-phase fraction and survival benefit from adjuvant chemotherapy or radiotherapy of breast cancer

O. Stål[1], L. Skoog[2], L.E. Rutqvist[3], J.M. Carstensen[4], S. Wingren[1], S. Sullivan[1], C. Andersson[1], M. Dufmats[1] & B. Nordenskjöld[1]

[1]*Department of Oncology, Faculty of Health Sciences, Linköping University, S-581 85 Linköping, Sweden;* [2]*Division of Cytology and the* [3]*Oncologic Centre, Karolinska Hospital, S-104 01 Stockholm, Sweden;* [4]*Department of Health and Society, Linköping University, S-581 85 Linköping, Sweden.*

**Summary** Cancer chemotherapy interacts with cell proliferation, but data on the relationship between cancer cell replication and the effect of adjuvant chemotherapy are scarce. We have investigated the S-phase fractions of the primary tumour from premenopausal breast cancer patients who participated in a randomised trial comparing 12 cycles of polychemotherapy (CMF) with post-operative radiotherapy. DNA flow cytometry was performed on frozen tissues from 208 primary breast carcinomas, of which the S-phase fraction was estimated in 176 cases. There was a significantly higher benefit from CMF among patients with a high S-phase fraction ($P = 0.0033$). The relative risk of distant recurrence or death in the chemotherapy group as compared with the radiotherapy group was 0.19 for patients whose tumours had an S-phase fraction of 10% or over (95% CI 0.07–0.51) and 1.55 (0.88–2.73) for patients whose tumours showed lower S-phase levels. The interaction was still significant in multivariate analysis ($P = 0.0057$), including lymph node metastases, tumour size and oestrogen receptor content. We conclude that the benefit from adjuvant chemotherapy compared with radiotherapy is largely confined to patients with highly proliferative tumours.

It has become evident that patients with early breast cancer benefit from adjuvant systemic therapy (Early Breast Cancer Trialists' Collaborative Group, 1992). Combination chemotherapy given at the time of primary treatment can reduce the risk of death from breast cancer by 25% in young women. On the other hand, most patients will either remain disease free without or relapse despite such treatment. An increasing number of biological factors have been demonstrated to correlate with prognosis of patients with breast cancer, but it is still difficult to predict accurately the response to treatment.

Cancer chemotherapy interacts with cell proliferation. Studies concerning treatment of metastatic breast cancer and preoperative chemotherapy have shown that patients with highly proliferating breast tumours are more likely to respond to cytotoxic therapy (Sulkes *et al.*, 1979; O'Reilly *et al.*, 1992; Remvikos *et al.*, 1992; Spyratos *et al.*, 1992). Data on the relationship between the proliferating activity of the primary tumour and the effect of adjuvant chemotherapy in patients with breast cancer are scarce and apparently conflicting (Bonadonna *et al.*, 1986; Hedley *et al.*, 1987; O'Reilly *et al.*, 1990; Witzig *et al.*, 1993).

We have therefore analysed retrospectively by DNA flow cytometry the S-phase fractions of frozen tumour specimens from premenopausal breast cancer patients who participated in a large randomised trial comparing 12 cycles of adjuvant polychemotherapy with post-operative radiotherapy.

### Patients and methods

In 1976 the Stockholm Breast Cancer Group initiated a trial to compare post-operative radiotherapy with adjuvant chemotherapy (Rutqvist *et al.*, 1989). The trial included pre- and post-menopausal patients with unilateral, operable breast cancer. Surgery consisted of modified radical mastectomy. The patients were required to have either histologically verified lymph node metastases or a tumour diameter, measured on the surgical specimen, exceeding 30 mm. Patient accrual started in November 1976 and ended in April 1990. The post-menopausal patients were also included in a concurrent comparison of adjuvant tamoxifen versus no adjuvant endocrine treatment.

Correspondence: O. Stål.
Received 25 March 1994; and in revised form 6 June 1994.

### *Radiotherapy and chemotherapy*

Radiotherapy was given with a high-voltage technique (Rutqvist *et al.*, 1989). The dose was 46 Gy with 2 Gy per fraction 5 days a week for total treatment time of about 4.5 weeks. The target volume included the chest wall, axilla, supraclavicular fossa and internal mammary nodes. The treatment intent was pursued in 98% of the patients randomised to radiotherapy. The chemotherapy protocol was the same as in the first Milan trial, that is 12 courses of a combination of cyclophosphamide, methotrexate and 5-fluorouracil (CMF). In the chemotherapy group, 9% did not receive the allocated treatment. Among patients who received at least one course of chemotherapy, there was a large difference in actual dose levels between the pre- and post-menopausal groups; only 15% of the post-menopausal patients received 85% or more of the planned dose compared with 38% in the premenopausal group.

### *Follow-up*

There was no significant difference in overall survival in any of the menopausal groups or when all patients were analysed (Rutqvist *et al.*, 1989). However, post-menopausal patients randomised to radiotherapy showed a lower incidence of both distant metastasis ($P<0.01$) and locoregional recurrence ($P<0.001$). In premenopausal patients, on the other hand, there was a tendency towards a lower risk of distant failure in the chemotherapy group ($P = 0.08$). In a recent update the difference was statistically significant (unpublished data).

The present study is restricted to the premenopausal group. From the 545 premenopausal patients included in the trial, frozen tumour samples that were spared after hormone receptor analysis were available in 208 cases. There was no significant difference in the distribution of lymph node status, tumour size or oestrogen receptor content comparing the selected cases with the original series. Eighty-five of the 208 patients had a distant recurrence or died during a median follow-up period of 8 years.

### *DNA flow cytometry*

A piece of the tumour specimen was minced in citrate buffer and afterwards a mixture of chicken and trout red blood cells was added as internal marker cells. A suspension of isolated nuclei was prepared without washing steps as described by

Vindelöv *et al.* (1983). The procedure included treatment with a detergent (0.1% NP-40), trypsin and RNAse followed by filtration through a 41 mm nylon mesh. The suspension was stained with propidium iodide and measured within 1 h. In addition, an imprint from the tissues was stained and examined to ascertain the presence of tumour cells in the sample. Cell suspensions were analysed with a FACScan flow cytometer (Becton Dickinson) equipped with a 15 mW argon laser. The Cellfit software (Becton Dickinson) was used for data acquisition and 15,000 events were recorded in each run.

DNA indices (DI) were calculated after zero point adjustment by using the chicken and trout red blood cells as internal controls. These showed 35% and 80% respectively of the fluorescence intensity of human (female) diploid cells stained with propidium iodide. Tumours with a single $G_{0/1}$ peak were classified as DNA diploid. If an additional peak was present the tumour was classified as DNA aneuploid. DNA aneuploid tumours with a DI <1.0 were called DNA hypodiploid. Small aneuploid populations, with a minimum of 1,000 cells, were separated from artefacts or diploid $G_2$/M cells by looking for a corresponding $G_2$/M peak. Median CV for tumour $G_{0/1}$ peaks was 3.6% with a range between 2.4% and 7%. For the estimation of S-phase fraction (SPF) a planimetric method was used, assuming the S-compartment to have rectangular distribution (Baisch *et al.*, 1975). In order to minimise the influence of overlapping populations in the DNA histogram, the height of the rectangle was estimated from a manually selected area that was judged as representative. Although the majority of the histograms showed only slight background debris to the right of the $G_2$/M peak, background correction was performed according to a baseline model. By this correction the mean number of histogram events per channel in an interval to the right of the $G_2$/M peak was subtracted from that in the S-phase interval. The SPF was estimated in 176 of the tumours (85%) and ranged between 0.5% and 35%. The mean S-phase value was 7.1% and the median was 5.9%.

### *Statistical methods*

The risk of distant recurrence or death in relation to different variables or interactions between variables was estimated and tested using Cox's proportional hazards model (Cox, 1972). The statistical significance of the interaction between S-phase fraction and treatment was evaluated by treating the S-phase fraction as a continuous variable in the Cox model, in that the prespecified S-phase categories, <5%, 5–10% and >10%, were coded 1, 2 and 3 respectively. To adjust for the potentially confounding effects of other variables, e.g. tumour stage and oestrogen receptor content, we performed additional Cox analyses in which the model was stratified according to the other variables. The product-limit method was used for estimation of cumulative probabilities of distant disease-free survival (Kaplan & Meier, 1958).

### Results

Characteristics of the material are shown in Table I. A high S-phase fraction was significantly associated with low oestrogen receptor content ($P$<0.0001) and DNA aneuploidy ($P$<0.0001), while subgroups categorised by tumour stage showed no significant difference according to SPF ($P$ = 0.18).

Using Cox's proportional hazards model, there was a clearly significant interaction between S-phase fraction and treatment effect (Table II). The relative risk of distant recurrence or death in the chemotherapy group as compared with the radiotherapy group was 0.19 for patients whose tumours had an S-phase fraction of 10% and over (95% confidence interval 0.07–0.51) and 1.55 (0.88–2.73) for patients whose tumours had an S-phase fraction less than 10%. For patients with highly proliferating tumours the estimated distant disease-free survival rate at 5 years was 68% in the CMF group, while it was 30% in the radiotherapy group (Figure 1). Similar results were obtained when restricting the analysis to survival (data not shown).

The correlation between S-phase fraction and the outcome difference between the two treatments was still significant in a stratified Cox analysis, taking into account possible interactons between treatment and the number of positive lymph nodes, tumour size and oestrogen receptor (ER) content (Table III). No other interaction was significant, although patients with oestrogen receptor negative or large tumours tended to benefit from chemotherapy. Restricting the analysis to ER-negative cases showed that those with a high S-phase fraction still did benefit from adjuvant CMF [relative risk: SPF ⩾10%, 0.12 (0.03–0.46); SPF<10%, 1.2 (0.49–2.9)], and the interaction between treatment and SPF was

**Table I** S-phase fraction in relation to tumour stage, oestrogen receptor content and DNA ploidy

| | *S-phase fraction* <5% (n = 74) | 5–10% (n = 62) | ⩾10% (n = 40) | *Total* (n = 176) |
|---|---|---|---|---|
| Nodal status, tumour size | | | | |
| N0, >30 mm | 5 (29)[a] | 7 (41) | 5 (29) | 17 (100) |
| N+, ⩽20 mm | 32 (52) | 20 (32) | 10 (16) | 62 (100) |
| N+, 21–30 mm | 22 (42) | 18 (34) | 13 (25) | 53 (100) |
| N+, >30 mm | 15 (34) | 17 (39) | 12 (27) | 44 (100) |
| ER content[b] | | | | |
| <0.1 fmol $\mu g^{-1}$ DNA | 16 (25) | 23 (36) | 25 (39) | 64 (100) |
| ⩾0.1 fmol $\mu g^{-1}$ DNA | 58 (53) | 37 (34) | 14 (13) | 109 (100) |
| DNA ploidy | | | | |
| Diploid | 54 (68) | 22 (28) | 3 (4) | 79 (100) |
| Aneuploid | 20 (21) | 40 (41) | 37 (38) | 97 (100) |

[a]Number of patients (%). [b]Data on ER content were missing in three cases.

**Table II** Relative risk of distant recurrence or death in the chemotherapy group compared with the radiotherapy group in relation to the S-phase fraction

| *SPF (%)* | *Number of patients[a] (%)* *Chemotherapy* | *Radiotherapy* | *Relative risk* | *95% confidence interval* | *Test for trend* |
|---|---|---|---|---|---|
| <5 | 37 (14) | 37 (11) | 1.30 | 0.59–2.87 | $\chi^2$ = 8.63 |
| 5–10 | 33 (19) | 29 (9) | 1.76 | 0.79–3.88 | d.f. = 1 |
| ⩾10 | 21 (6) | 19 (14) | 0.19 | 0.07–0.51 | $P$ = 0.0033 |

[a]Number of distant recurrences or deaths is shown within parentheses.

**Figure 1** Distant disease-free survival of patients treated with chemotherapy (CMF) compared with the radiotherapy group (RT) in relation to the S-phase fraction. Numbers of patients and distant recurrences or deaths are presented within parentheses.

significant ($P = 0.0030$). A similar trend was seen for the ER-positive subgroup.

In the DNA diploid and the DNA aneuploid subgroups, there was no significant difference in outcome between patients receiving chemotherapy and radiotherapy (Table IV). The relative risk comparing the two treatments was not significantly different for patients having DNA diploid or aneuploid tumours ($P = 0.63$). If the tumours were categorised into DNA hypodiploids (11%) and others, we found a significant interaction with treatment effect ($P = 0.0012$). Among the patients with DNA hypodiploid tumours, all eight patients receiving radiotherapy relapsed or died during the follow-up period compared with 5 of 14 patients receiving cytotoxic therapy. Of the DNA hypodiploid tumours with assessable S-phase fraction, most tumours (72%) showed a level of 10% or higher.

### Discussion

The results of the present study suggest that the benefit from adjuvant chemotherapy in premenopausal breast cancer patients is largely confined to those with highly proliferative primary tumours. Previously reported evidence for this is scarce for adjuvant chemotherapy, but the presumption that rapidly proliferating tumours are more chemosensitive than others is supported by studies concerning preoperative chemotherapy and the treatment of metastatic disease. Sulkes *et al.* (1979) analysed the response to chemotherapy in 25 women with disseminated breast cancers in relation to the proliferative activity estimated as the thymidine labelling index (TLI). The difference in response was strongly correlated to TLI. All nine responders showed an LI of 9% or greater compared with 5 of the 16 who did not respond. Furthermore, tumour regression in primary breast cancer observed after preoperative chemotherapy has been significantly associated with high S-phase fraction (O'Reilly *et al.*, 1992; Remvikos *et al.*, 1992; Spyratos *et al.*, 1992), and repeated cytological sampling during chemotherapy has indicated that tumour regression is parallelled by a decreasing growth fraction (Skoog *et al.*, 1992).

In trials of adjuvant chemotherapy, the effect of treatment related to SPF and other factors have been investigated, but clear conclusions from these reports have not been made. Hedley *et al* (1987) were not able to identify a subgroup defined by DNA flow cytometry in whom adjuvant treatment was especially beneficial. Witzig *et al.* (1993) reported that DNA aneuploidy, but not a high SPF, identified a subgroup of post-menopausal patients who tended to benefit from adjuvant chemotherapy. O'Reilly *et al.* (1990) presented data on 69 premenopausal patients randomised to receive either no adjuvant therapy or CMF. The treatment significantly improved the recurrence-free survival for patients with high tumour SPF values as well as for patients with low S-phase

**Table III** Adjusted relative risk of distant recurrence or death in the chemotherapy group compared with the radiotherapy group in relation to the S-phase fraction, tumour stage and oestrogen receptor status

| | *Number of patients[a] (%)* Chemotherapy | Radiotherapy | *Adjusted relative risk[b]* | *95% confidence interval* | *Test for heterogeneity* |
|---|---|---|---|---|---|
| SPF (%) | | | | | |
| <5 | 37 (14) | 37 (11) | 1.39 | 0.60-3.23 | $\chi^2 = 7.64^c$ |
| 5-10 | 32 (18) | 28 (9) | 1.28 | 0.52-31.6 | d.f. = 1 |
| ≥10 | 20 (6) | 19 (14) | 0.21 | 0.07-0.51 | $P = 0.0057$ |
| Tumour stage | | | | | |
| N0, >30 mm | 11 (3) | 5 (3) | 0.21 | 0.04-1.17 | |
| N+, <21 mm | 26 (11) | 36 (11) | 1.55 | 0.66-3.65 | $\chi^2 = 4.09$ |
| N+, 21-30 mm | 28 (10) | 24 (7) | 0.84 | 0.31-2.29 | d.f. = 3 |
| N+, >30 mm | 24 (14) | 19 (13) | 0.82 | 0.36-1.86 | $P = 0.25$ |
| ER content (fmol $\mu g^{-1}$ DNA) | | | | | |
| <0.1 | 36 (15) | 28 (17) | 0.63 | 0.28-1.41 | $\chi^2 = 0.71$ |
| ≥0.1 | 53 (23) | 56 (17) | 1.00 | 0.50-2.02 | $P = 0.40$ |

[a]Number of distant recurrences or deaths is shown within parentheses. [b]Adjusted for the other variables listed in the table. [c]Test for trend.

**Table IV** Relative risk of distant recurrence or death in the chemotherapy group compared with the radiotherapy group in relation to DNA ploidy

| *DNA ploidy* | *Number of patients[a] (%)* Chemotherapy | Radiotherapy | *Relative risk* | *95% confidence interval* | *Test for heterogeneity* |
|---|---|---|---|---|---|
| Diploid | 43 (16) | 37 (13) | 0.99 | 0.47-2.08 | $\chi^2 = 0.23$ |
| Aneuploid | 67 (28) | 61 (28) | 0.78 | 0.45-1.32 | $P = 0.63$ |

[a]Number of distant recurrences or deaths is shown within parentheses.

levels. The same was observed if the patients were divided by tumour grade, in contrast to the study of Fisher *et al.* (1983), who found that a favourable response to adjuvant therapy was more pronounced in patients with poorly differentiated tumours. Similar to the present results, however, Bonadonna *et al.* (1986) found that the benefit from adjuvant CMF was largely confined to patients with rapidly proliferating tumours. In both studies a strong interaction between SPF and CMF response was seen among ER-negative patients, suggesting that the mechanism of action of chemotherapy was cytotoxicity rather than chemical castration, which has been proposed as a possible mechanism in premenopausal patients. (Brincker *et al.*, 1987).

Factors other than proliferative activity have been investigated for their possible interaction with response to chemotherapy. Gusterson *et al.* (1992) observed a trend towards less responsiveness in tumours overexpressing the c-*erb*B-2 oncogene. In studies of preoperative treatment, a more favourable response was found if the tumour was poorly differentiated, oestrogen receptor negative or DNA aneuploid (Neville *et al.*, 1992; Spyratos *et al.*, 1992). These factors are known to correlate with S-phase fraction. In the series of Remvikos *et al.* (1989), SPF was a better predictor of tumour responsiveness than tumour grade, hormone receptor content or DNA ploidy. In line with these results, we found that S-phase fraction correlated with response when adjusting for other variables in multivariate analysis. DNA hypodiploid tumours, which have shown to be associated with low age (Stål *et al.*, 1992) and poor prognosis (Fernö *et al.*, 1992) in breast cancer, also seemed to be more chemosensitive than others. A clear conclusion about an independent value cannot be made because most of the DNA hypodiploid tumours showed a high S-phase fraction.

The hypothesis that the proliferative activity of the tumour might be related to the response to chemotherapy is attractive, since most cytotoxic agents act specifically against actively DNA replicating cells. Techniques used to estimate proliferative activity are, however, associated with methodological problems. It should be noted that the investigators who found a significant relation between cell proliferation and response to treatment used either the thymidine labelling technique (Sulkes *et al.*, 1979; Bonadonna *et al.*, 1986) or DNA flow cytometry on fresh or frozen material (Remvikos *et al.*, 1989; O'Reilly *et al.*, 1992; Spyratos *et al.*, 1992), in contrast to others who employed paraffin-embedded fixed tissue (Hedley *et al.*, 1987; O'Reilly *et al.*, 1990; Witzig *et al.*, 1993). Irrespective of the starting material used, other problems concern intratumoral heterogeneity and errors related to the calculation model for SPF. However, although different models produce different estimated levels of SPF, we and other investigators have observed that a significant prognostic value is obtained with several models, including the manual rectangular method (Joensuu *et al.*, 1994; Wingren *et al.*, 1994). An indirect support for an interaction between SPF and response to adjuvant chemotherapy was reported from a study in which all the patients received adjuvant treatment (Kute *et al.*, 1990). It was found that those with a tumour of high SPF tended to have a better prognosis in contrast to the prediction of a high recurrence risk mostly found in series of systemically untreated patients.

In our study the effect of CMF was compared with that of post-operative radiotherapy in contrast to trials with adjuvant chemotherapy in which the control patients received no adjuvant treatment. Unlike the results obtained for premenopausal patients by O'Reilly *et al.* (1990), we found no benefit from CMF for patients with low S-phase levels, but rather a trend towards longer distant disease-free survival in the radiotherapy group. Radiotherapy has long been regarded as locoregional treatment with little or no effect on distant recurrence or survival. However, a significant reduction in distant metastases with adjuvant radiotherapy compared with surgery alone has been observed among node-positive patients (Auquier *et al.*, 1992). Although a subsequent trial has shown that the risk of distant recurrence in premenopausal patients is further reduced if radiotherapy is replaced by CMF (Rutqvist *et al.*, 1989), we cannot exclude the possibility of a minor benefit from chemotherapy compared with no post-operative treatment among patients whose tumours show low proliferative activity. Compared with post-operative radiotherapy, we conclude that the benefit from adjuvant chemotherapy is largely confined to patients with highly proliferative primary tumours. In this view, it is promising that a high proportion of relapses in systemically untreated premenopausal patients seems to be identified by S-phase fraction (Stål *et al.*, 1992). Recently, we also reported results suggesting that SPF is a long-term prognostic factor in small node-negative tumours (T1N0) (Stål *et al.*, 1993). These results, together with the present data, suggest that estimation of proliferative activity could facilitate the identification of early breast cancer patients suitable for adjuvant chemotherapy.

This work was supported by grants from the Swedish Cancer Society.

### References

AUQUIER, A., RUTQVIST, L.E., HØST, H., ROTSTEIN, S. & ARRIAGADA, R. (1992). Post-mastectomy megavoltage radiotherapy: the Oslo and Stockholm trials. *Eur. J. Cancer*, **28**, 433-437.

BAISCH, H., GÖHDE, W. & LINDEN, W.A. (1975). Analysis of PCP-data to determine the fraction of cells in the various phases of the cell cycle. *Radiat-Environ. Biophys.*, **12**, 31-39.

BONADONNA, G., VALAGUSSA, P., TANCINI, G., ROSSI, A., BRAMBILLA, C., ZAMBETTI, M., BIGNAMI, P., DI FRONZO, G. & SILVESTRINI, R. (1986). Current status of Milan adjuvant chemotherapy trials for node-positive and node-negative breast cancer. *Natl Cancer Inst. Monogr.*, **1**, 45-49.

BRINCKER, H., ROSE, C., RANK, F., MOURIDSEN, H.T., JAKOBSEN, P., DOMBERNOWSKY, P., PANDURO, J. & ANDERSEN, K.W. (1987). Evidence of a castration-mediated effect of adjuvant cytotoxic chemotherapy in premenopausal breast cancer. *J. Clin. Oncol.*, **5**, 1771-1778.

COX, D.R. (1972). Regression models and life tables (with discussion). *J. Stat. Soc. B.*, **34**, 187-220.

EARLY BREAST CANCER TRIALISTS' COLLABORATIVE GROUP (1992). Systemic treatment of early breast cancer by hormonal, cytotoxic, or immune therapy. 133 randomised trials involving 31,000 recurrences and 23,000 deaths among 75,000 women. *Lancet*, **339**, 1-15, 71-85.

FERNÖ, M., BALDETORP, B., BORG, Å., OLSSON, H., SIGURDSSON, H. & KILLANDER, D. (1992). Flow cytometric DNA index and S-phase fraction in breast cancer in relation to other prognostic variables and to clinical outcome. *Acta Oncol.*, **31**, 157-165.

FISHER, E.R., REDMOND, C., FISHER, B. & PARTICIPATING NSABP INVESTIGATORS (1983). Pathologic findings from the National Surgical Adjuvant Breast Project. VIII. Relationship of chemotherapeutic responsiveness to tumor differentiation. *Cancer*, **51**, 181-191.

GUSTERSON, B.A., GELBER, R.D., GOLDHIRSCH, A., PRICE, K.N., SÄVE-SÖLDERBORGH, J., ANBAZHAGAN, R., STYLES, J., RUDENSTAM, C.-M., GOLOUH, R., REED, R., MARTINEZ-TELLO, F., TILTMAN, A., TORHORST, J., GRIGOLATO, P., BETTELHEIM, R., NEVILLE, A.M., BÜRKI, K., CASTIGLIONE, M., COLLINS, J., LINDTNER, J. & SENN, H.-J. FOR THE INTERNATIONAL (LUDWIG) BREAST CANCER STUDY GROUP (1992). Prognostic importance of c-erB-2 expression in breast cancer. *J. Clin. Oncol.*, **10**, 1049-1056.

HEDLEY, D.W., RUGG, C.A. & GELBER, R.D. (1987). Association of DNA index and S-phase fraction with prognosis of nodes positive early breast cancer. *Cancer Res.*, **47**, 4729-4735.

JOENSUU, H., ELOMAA, L., KLEMI, P.J. & TOIKANNEN, S. (1994). Comparison of five methods to calculate the S phase fraction size. Abstract 252. *Anal. Cell. Pathol.*, **6**, 263.

KAPLAN, E. & MEIER, P. (1958). Non parametric estimation from incomplete observations. *J. Am. Stat. Assoc.*, **53**, 457–481.

KUTE, T.E., MUSS, H.B., COOPER, M.R., CASE, L.D., BUSS, D., STANLEY, V., GREGORY, B., GALLESHOW, J. & BOOHER, K. (1990). The use of flow cytometry for the prognosis of stage II adjuvant treated breast cancer patients. *Cancer*, **66**, 1810–1816.

NEVILLE, A.M., BETTELHEIM, R., GELBER, R.D., SÄVE-SÖDERBERGH, J., DAVIS, B.W., REED, R., TORHORST, J., GOLOUH, R., PETERSON, H.F., PRICE, K.N., ISLEY, M., RUDENSTAM, C.-M., COLLINS, J., CASTIGLIONE, M., SENN, H.-J. & GOLDHIRSCH, A. FOR THE INTERNATIONAL (LUDWIG) BREAST CANCER STUDY GROUP (1992). Factors predicting treatment responsiveness and prognosis in node-negative breast cancer. *J. Clin. Oncol.*, **10**, 696–705.

O'REILLY, S.M., CAMPLEJOHN, R.S., MILLIS, R.R., RUBENS, R.D. & RICHARDS, M.A. (1990). Proliferative activity, histological grade and benefit from adjuvant chemotherapy in node positive breast cancer. *Eur. J. Cancer*, **26**, 1035–1038.

O'REILLY, S.M., CAMPLEJOHN, R.S., RUBENS, R.D. & RICHARDS, M.A. (1992). DNA flow cytometry and response to preoperative chemotherapy for primary breast cancer. *Eur. J. Cancer*, **28**, 681–683.

REMVIKOS, Y., BEUZEBOC, P., ZAJDELA, A., VOILLEMOT, N., MAGDELENAT, H. & POUILLART, P. (1989). Correlation of pretreatment proliferative activity of breast cancer with the response to cytotoxic chemotherapy. *J. Natl Cancer Inst.*, **81**, 1383–1387.

RUTVIST, L.E., CEDERMARK, B., GLAS, U., JOHANSSON, H., ROTSTEIN, S., SKOOG, L., SOMELL, A., THEVE, T., ASKERGREN, J., FRIBERG, S., BERGSTRÖM, J., BLOMSTEDT, B., RÄF, L., SILFVERSWÄRD, C. & EINHORN, J. (1989). Radiotherapy, chemotherapy, and tamoxifen as adjuncts to surgery in early breast cancer: a summary of three randomized trials. *Int. J. Radiat. Oncol. Biol. Phys.*, **16**, 629–639.

SKOOG, L., RUTQVIST, L.E., WILKING, N. (1992). Analysis of hormone receptors and proliferation fraction in fine-needle aspirates from primary breast carcinomas during chemotherapy or tamoxifen treatment. *Acta Oncol.*, **31**, 139–141.

SPYRATOS, F., BRIFFOD, M., TUBIANA-HULIN, M., ANDRIEU, C., MAYRAS, C., PALLUD, C., LASRY, S. & ROUESSÉ, J. (1992). Sequential cytopunctures during preoperative chemotherapy for primary breast carcinoma. II. DNA flow cytometry changes during chemotherapy, tumor regression, and short-term follow-up. *Cancer*, **69**, 470–475.

STÅL, O., CARSTENSEN, J., HATSCHEK, T. & NORDENSKJÖLD, B. (1992). Significance of S-phase fraction and hormone receptor content in the management of young breast cancer patients. *Br. J. Cancer*, **66**, 706–711.

STÅL, O., DUFMATS, M., HATSCHEK, T., CARSTENSEN, J., KLINTENBERG, C., RUTQVIST, L.-E., SKOOG, L., SULLIVAN, S., WINGREN, S. & NORDENSKJÖLD, B. (1993). S-phase fraction is a prognostic factor in stage I breast carcinoma. *J. Clin. Oncol.*, **11**, 1717–1722.

SULKES, A., LIVINGSTON, R.B. & MURPHY, W.K. (1979). Tritiated thymidine labeling index and response in human breast cancer. *J. Natl Cancer Inst.*, **62**, 513–515.

VINDELÖV, L.L., CHRISTENSEN, I.J. & NISSEN, N.I. (1983). A detergent-trypsin method for the preparation of nuclei for flow cytometric DNA analysis. *Cytometry*, **3**, 323–327.

WINGREN, S., STÅL, O., CARSTENSEN, J., SUN, X.-F. & NORDENSKJÖLD, B. (1994). S-phase determination of immunoselected cytokeratin-containing breast cancer cells improves the prediction of recurrence. *Breast Cancer Res. Treat.*, **29**, 179–187.

WITZIG, T.E., INGLE, J.N., SCHAID, D.J., WOLD, L.E., BARLOW, J.F., GONCHOROFF, N.J., GERSTNER, J.B., KROOK, J.E., GRANT, C.S. & KATZMANN, J.A. (1993). DNA ploidy and percent S-phase as prognostic factors in node-positive breast cancer: results from patients enrolled in two prospective randomized trials. *J. Clin. Oncol.*, **11**, 351–359.


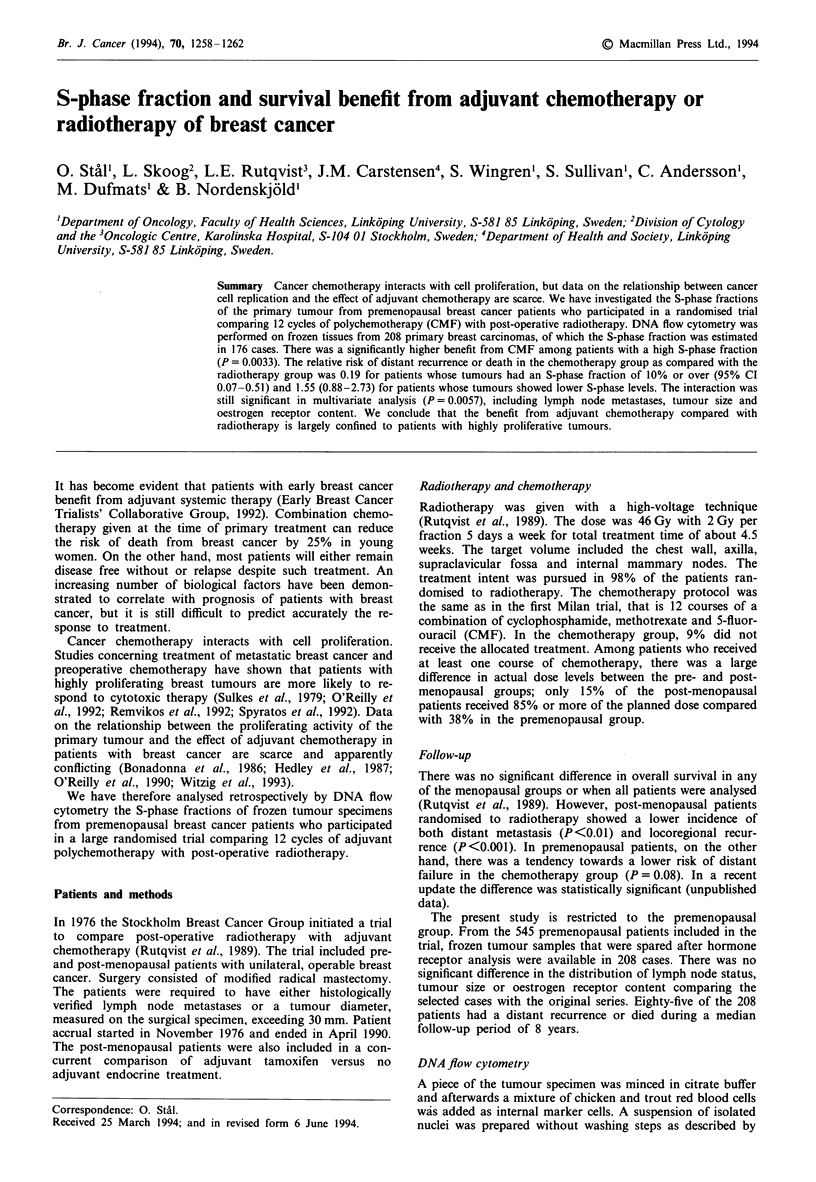

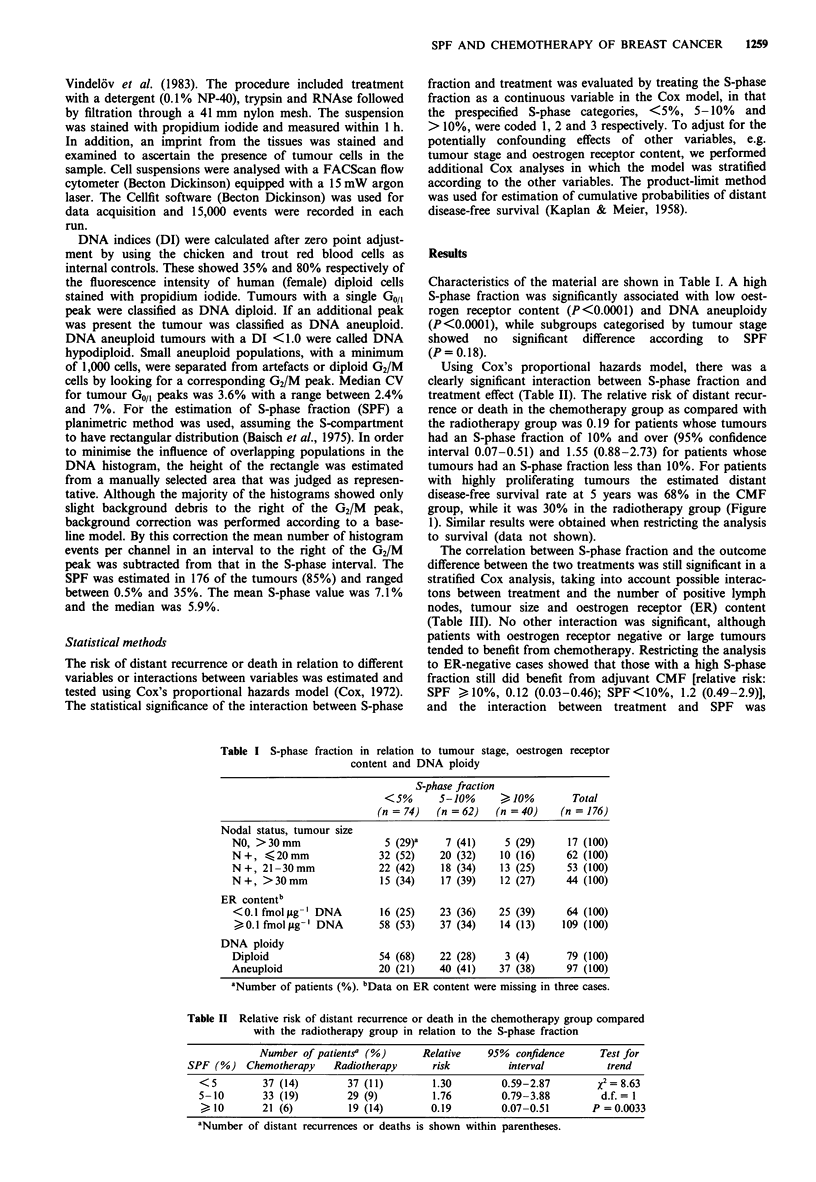

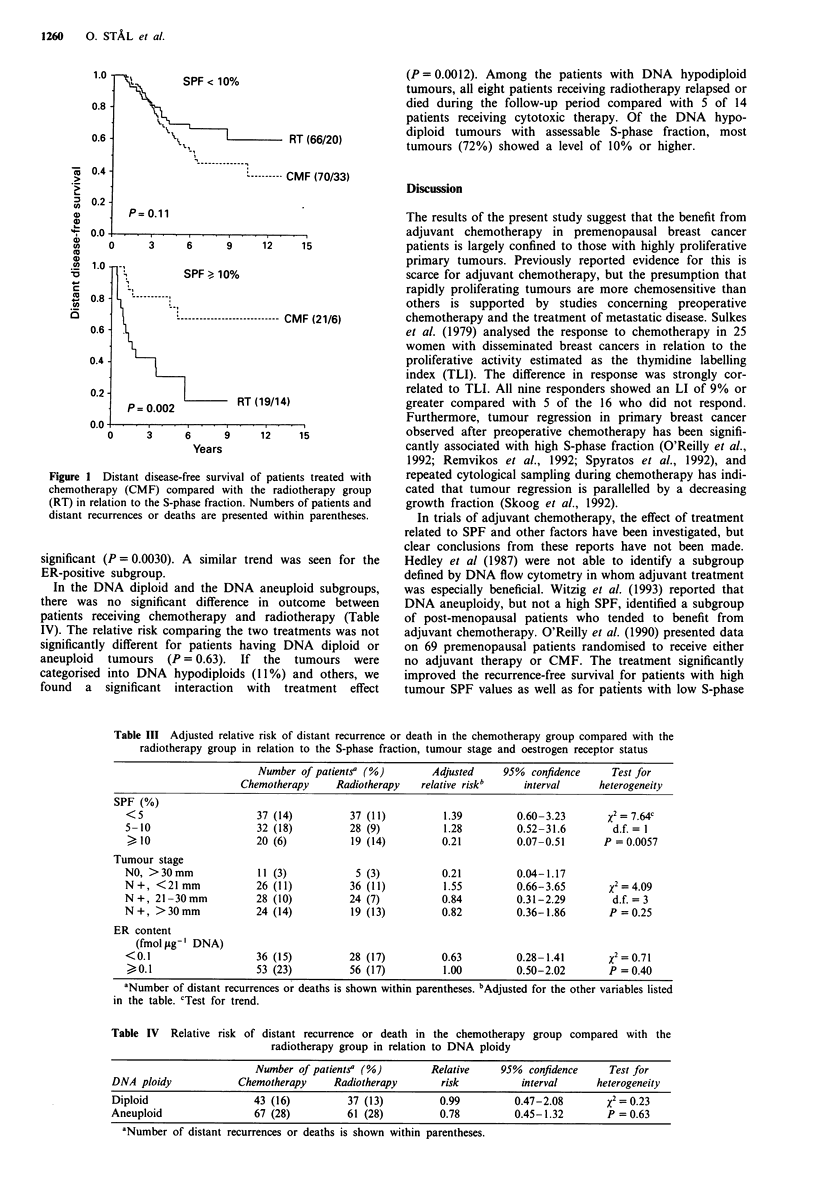

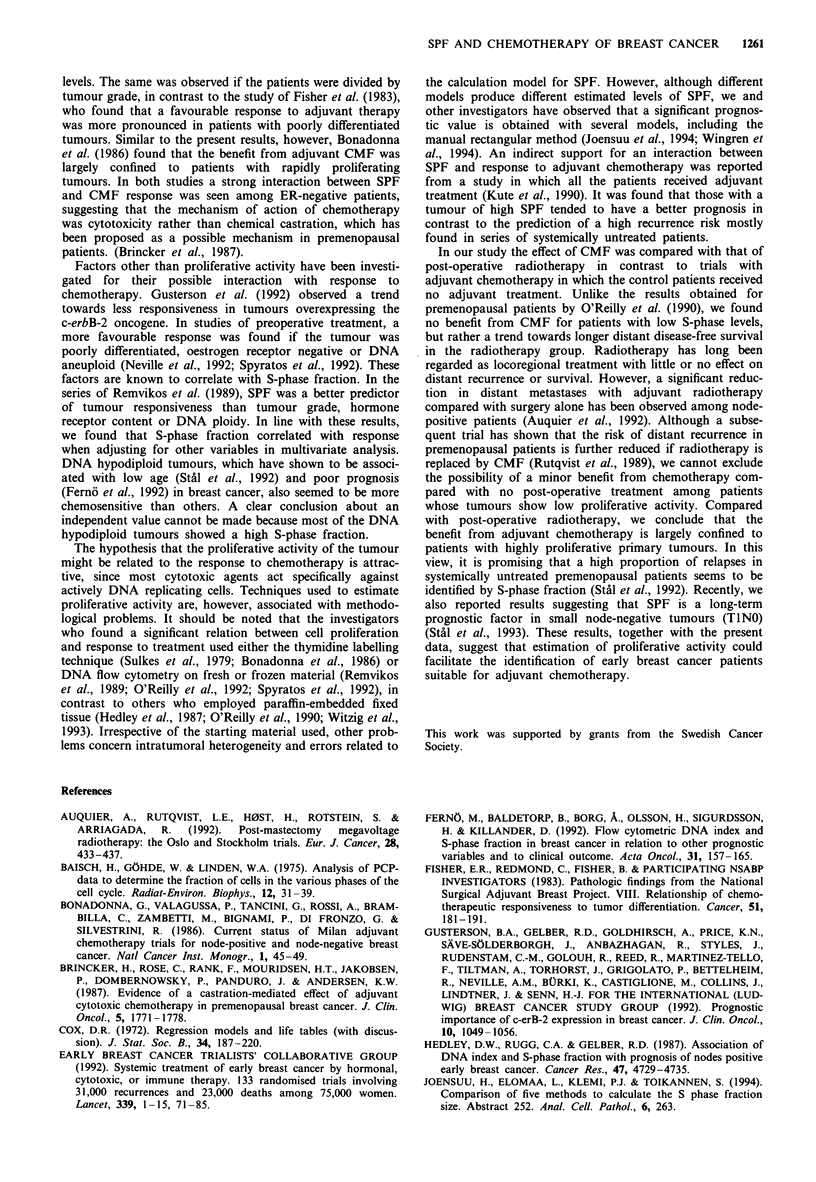

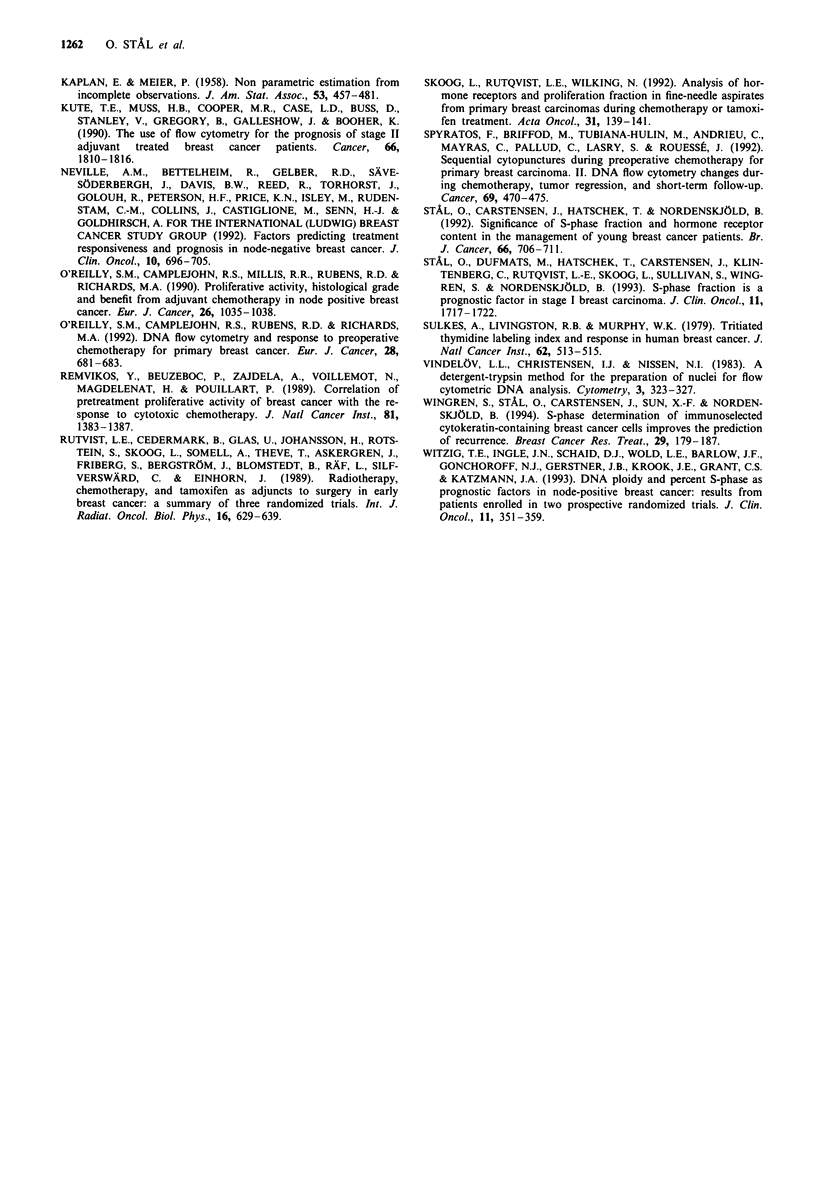


## References

[OCR_00518] Auquier A., Rutqvist L. E., Høst H., Rotstein S., Arriagada R. (1992). Post-mastectomy megavoltage radiotherapy: the Oslo and Stockholm trials.. Eur J Cancer.

[OCR_00524] Baisch H., Göhde W., Linden W. A. (1975). Analysis of PCP-data to determine the fraction of cells in the various phases of cell cycle.. Radiat Environ Biophys.

[OCR_00531] Bonadonna G., Valagussa P., Tancini G., Rossi A., Brambilla C., Zambetti M., Bignami P., Di Fronzo G., Silvestrini R. (1986). Current status of Milan adjuvant chemotherapy trials for node-positive and node-negative breast cancer.. NCI Monogr.

[OCR_00536] Brincker H., Rose C., Rank F., Mouridsen H. T., Jakobsen A., Dombernowsky P., Panduro J., Andersen K. W. (1987). Evidence of a castration-mediated effect of adjuvant cytotoxic chemotherapy in premenopausal breast cancer.. J Clin Oncol.

[OCR_00554] Fernö M., Baldetorp B., Borg A., Olsson H., Sigurdsson H., Killander D. (1992). Flow cytometric DNA index and S-phase fraction in breast cancer in relation to other prognostic variables and to clinical outcome.. Acta Oncol.

[OCR_00562] Fisher E. R., Redmond C., Fisher B. (1983). Pathologic findings from the National Surgical Adjuvant Breast Project. VIII. Relationship of chemotherapeutic responsiveness to tumor differentiation.. Cancer.

[OCR_00578] Hedley D. W., Rugg C. A., Gelber R. D. (1987). Association of DNA index and S-phase fraction with prognosis of nodes positive early breast cancer.. Cancer Res.

[OCR_00594] Kute T. E., Muss H. B., Cooper M. R., Case L. D., Buss D., Stanley V., Gregory B., Galleshaw J., Booher K. (1990). The use of flow cytometry for the prognosis of stage II adjuvant treated breast cancer patients.. Cancer.

[OCR_00601] Neville A. M., Bettelheim R., Gelber R. D., Säve-Söderbergh J., Davis B. W., Reed R., Torhorst J., Golouh R., Peterson H. F., Price K. N. (1992). Factors predicting treatment responsiveness and prognosis in node-negative breast cancer. The International (Ludwig) Breast Cancer Study Group.. J Clin Oncol.

[OCR_00611] O'Reilly S. M., Camplejohn R. S., Millis R. R., Rubens R. D., Richards M. A. (1990). Proliferative activity, histological grade and benefit from adjuvant chemotherapy in node positive breast cancer.. Eur J Cancer.

[OCR_00617] O'Reilly S. M., Camplejohn R. S., Rubens R. D., Richards M. A. (1992). DNA flow cytometry and response to preoperative chemotherapy for primary breast cancer.. Eur J Cancer.

[OCR_00623] Remvikos Y., Beuzeboc P., Zajdela A., Voillemot N., Magdelénat H., Pouillart P. (1989). Correlation of pretreatment proliferative activity of breast cancer with the response to cytotoxic chemotherapy.. J Natl Cancer Inst.

[OCR_00634] Rutqvist L. E., Cedermark B., Glas U., Johansson H., Rotstein S., Skoog L., Somell A., Theve T., Askergren J., Friberg S. (1989). Radiotherapy, chemotherapy, and tamoxifen as adjuncts to surgery in early breast cancer: a summary of three randomized trials.. Int J Radiat Oncol Biol Phys.

[OCR_00639] Skoog L., Rutqvist L. E., Wilking N. (1992). Analysis of hormone receptors and proliferation fraction in fine-needle aspirates from primary breast carcinomas during chemotherapy or tamoxifen treatment.. Acta Oncol.

[OCR_00645] Spyratos F., Briffod M., Tubiana-Hulin M., Andrieu C., Mayras C., Pallud C., Lasry S., Rouëssé J. (1992). Sequential cytopunctures during preoperative chemotherapy for primary breast carcinoma. II. DNA flow cytometry changes during chemotherapy, tumor regression, and short-term follow-up.. Cancer.

[OCR_00653] Stål O., Carstensen J., Hatschek T., Nordenskjöld B. (1992). Significance of S-phase fraction and hormone receptor content in the management of young breast cancer patients.. Br J Cancer.

[OCR_00662] Stål O., Dufmats M., Hatschek T., Carstensen J., Klintenberg C., Rutqvist L. E., Skoog L., Sullivan S., Wingren S., Nordenskjöld B. (1993). S-phase fraction is a prognostic factor in stage I breast carcinoma.. J Clin Oncol.

[OCR_00666] Sulkes A., Livingston R. B., Murphy W. K. (1979). Tritiated thymidine labeling index and response in human breast cancer.. J Natl Cancer Inst.

[OCR_00671] Vindeløv L. L., Christensen I. J., Nissen N. I. (1983). A detergent-trypsin method for the preparation of nuclei for flow cytometric DNA analysis.. Cytometry.

[OCR_00678] Wingren S., Stål O., Carstensen J., Sun X. F., Nordenskjöld B. (1994). S-phase determination of immunoselected cytokeratin-containing breast cancer cells improves the prediction of recurrence.. Breast Cancer Res Treat.

[OCR_00682] Witzig T. E., Ingle J. N., Schaid D. J., Wold L. E., Barlow J. F., Gonchoroff N. J., Gerstner J. B., Krook J. E., Grant C. S., Katzmann J. A. (1993). DNA ploidy and percent S-phase as prognostic factors in node-positive breast cancer: results from patients enrolled in two prospective randomized trials.. J Clin Oncol.

